# Pitfall of endoscopic ultrasound-guided rendezvous: sphincterotomy-related perforation caused by misidentification of the guidewire exit

**DOI:** 10.1055/a-2957-9893

**Published:** 2026-09-30

**Authors:** Satoshi Ito, Toru Okuzono, Sofumi Son, Riku Meguro, Tomoki Matsuda

**Affiliations:** 1 Department of Gastroenterology 26387 Sendai Kosei HospitalSendaiMiyagi PrefectureJapan

Although endoscopic ultrasound-guided rendezvous (EUS-RV) is useful for difficult biliary cannulation, adverse events have been reported. 1​ ​2​ We report retroperitoneal perforation caused by endoscopic sphincterotomy (EST) after misidentification of the guidewire exit.

An 83-year-old woman underwent endoscopic retrograde cholangiopancreatography (ERCP) for common bile duct stone removal ( Fig. 1 ). As biliary cannulation was unsuccessful in the presence of a periampullary diverticulum, EUS-RV was performed ( Video 1 ).

**Fig. 1:**
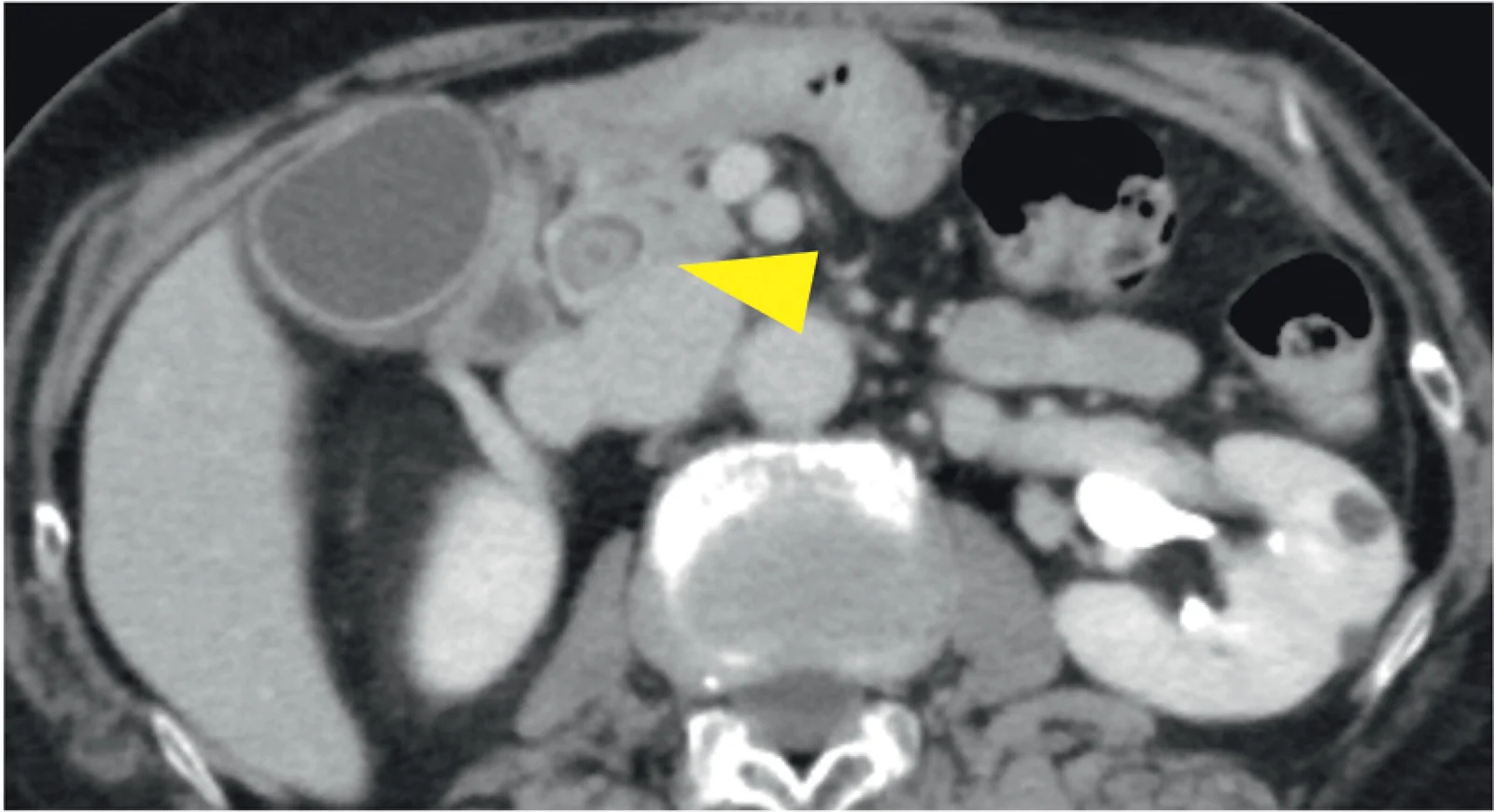
Computed tomography scan revealing common bile duct stones (yellow arrowhead).

**Video 1** Misidentification of the guidewire exit through a site other than the true papilla resulted in retroperitoneal perforation after endoscopic sphincterotomy during EUS-guided rendezvous.


Using a linear echoendoscope, the dilated B2 intrahepatic bile duct was transgastrically punctured with a 19-gauge needle. A guidewire was advanced toward the duodenum under fluoroscopy, and the echoendoscope was exchanged for a duodenoscope. The guidewire was grasped with forceps and pulled through the working channel. Incorrectly assuming that the guidewire exited through the native papilla, we advanced a sphincterotome over it and performed EST without additional cholangiography. Fluoroscopy demonstrated retroperitoneal air, indicating perforation. Stone extraction was abandoned, and an endoscopic retrograde biliary drainage stent and an endoscopic nasobiliary drainage tube were placed. Following conservative management ( Fig. 2a, b ), repeat ERCP demonstrated that the guidewire had exited through an iatrogenic fistulous tract into the periampullary diverticulum, rather than through the native papilla ( Fig. 3 ). This misidentification led to inadvertent EST and subsequent perforation. Two months later, balloon dilation of the fistulous tract enabled successful stone removal.

**Fig. 2:**
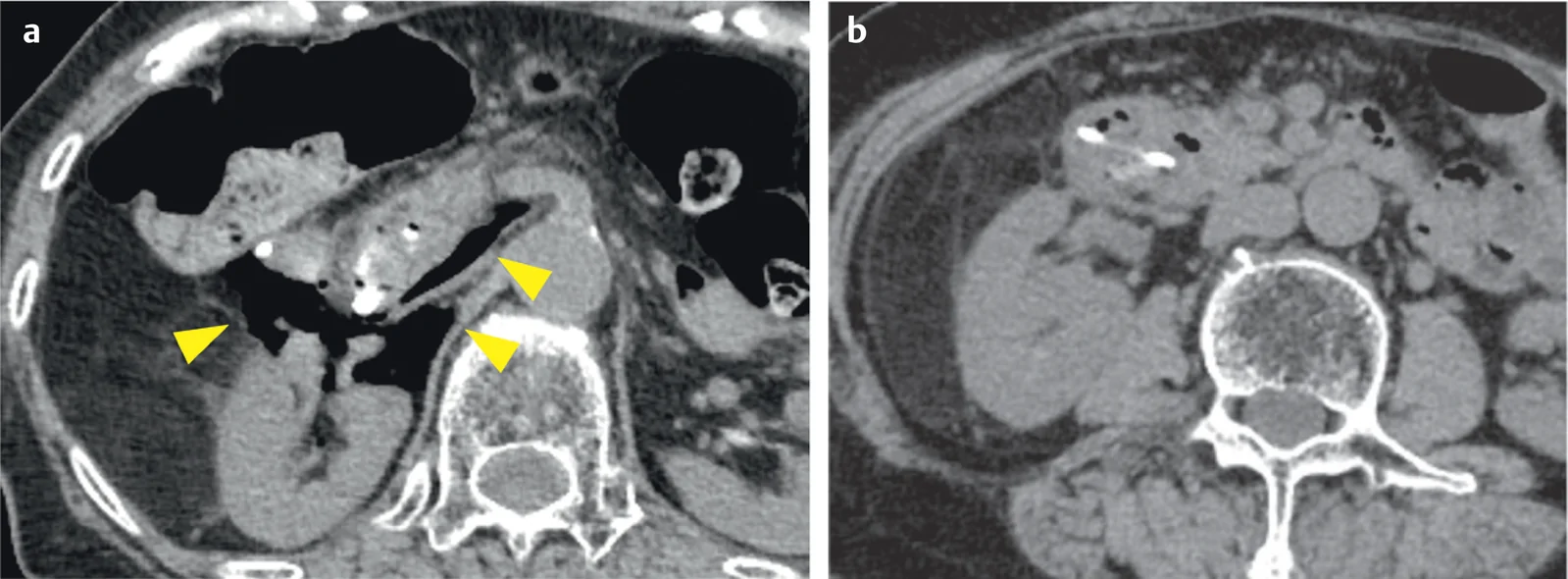
( **a** ) Computed tomographic scan after the procedure revealing retroperitoneal air (yellow arrowhead), indicating retroperitoneal perforation. ( **b** ) After conservative management, the retroperitoneal air resolved.

**Fig. 3:**
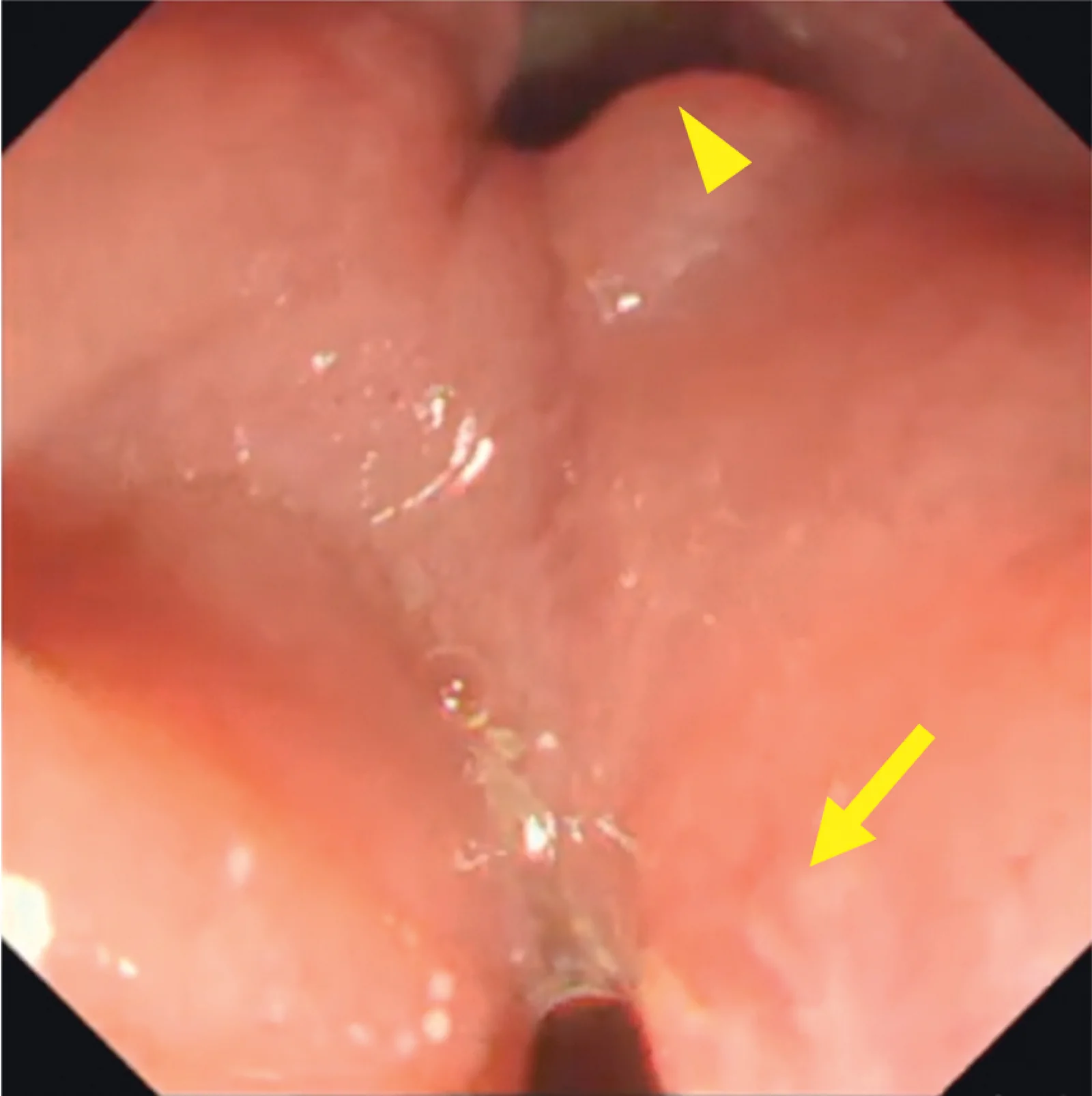
An endoscopic image showing the papilla (yellow arrow) and the iatrogenic fistulous tract (yellow arrowhead).

The impacted distal common bile duct stone may have prevented transpapillary guidewire passage, causing the guidewire to perforate the distal bile duct and enter the adjacent periampullary diverticulum. This case demonstrates that the guidewire may exit through an iatrogenic fistulous tract instead of the native papilla. Therefore, before EST, the guidewire exit and the course of the bile duct should be confirmed endoscopically and cholangiographically, respectively. If a non-native opening is suspected, temporary biliary drainage with a plastic stent alone should be considered until the fistulous tract has matured.

Endoscopy_UCTN_Code_CPL_1AL_2AD

## Declaration of GenAI Use

The authors used ElevenLabs, a generative artificial intelligence (AI)-based text-to-speech tool, solely to generate the English-language narration for the accompanying video from a script prepared by the authors. Generative AI was not used to prepare the manuscript or to generate or modify any scientific content, clinical images, video footage, or data. The authors reviewed and verified the narration and take full responsibility for the final manuscript and video.

## References

[1] Iwashita T Yasuda I Mukai T EUS-guided rendezvous for difficult biliary cannulation using a standardized algorithm: a multicenter prospective pilot study (with videos) Gastrointest Endos 2016 83 394 400 10.1016/j.gie.2015.04.043 26089103

[2] Klair J S Zafar Y Ashat M Effectiveness and safety of EUS rendezvous after failed biliary cannulation with ERCP: a systematic review and proportion meta-analysis J Clin Gastroenterol 2023 57 211 217 10.1097/MCG.0000000000001543 34009843

